# Downregulation of CAMK2N1 due to DNA Hypermethylation Mediated by DNMT1 that Promotes the Progression of Prostate Cancer

**DOI:** 10.1155/2023/4539045

**Published:** 2023-01-30

**Authors:** Wei Peng, Huan Feng, Linhao Pang, Junfeng Zhang, Yi Hao, Xian Wei, Qidong Xia, Zhewen Wei, Wen Song, Shaogang Wang, Jihong Liu, Ke Chen, Tao Wang

**Affiliations:** ^1^Department of Urology, Tongji Hospital, Tongji Medical College, Huazhong University of Science and Technology, Wuhan, China; ^2^Institute of Urology, Tongji Hospital, Tongji Medical College, Huazhong University of Science and Technology, Wuhan, China; ^3^Department of Urology, Suining Central Hospital, Chongqing Medical University, Suining, China; ^4^Department of Urology, The Central Hospital of Enshi Tujia and Miao Autonomous Prefecture, Enshi, China; ^5^Department of Pathogen Biology, School of Basic Medicine, Tongji Medical College, Huazhong University of Science and Technology, Wuhan, China

## Abstract

Calcium/calmodulin-dependentprotein kinase II inhibitor I (CAMK2N1) as one of the tumor suppressor genes is significantly downregulated in prostate cancer (PCa). Reduced expression of CAMK2N1 is positively correlated with PCa progression. However, the mechanisms of CAMK2N1 downregulation in PCa are still unclear. The promoter region of CAMK2N1 contains a large number of CG loci, providing the possibility for DNA methylation. Consequently, we hypothesized that DNA methylation can result in the reduced expression of CAMK2N1 in PCa. In the presented study, the DNA methylation level of CAMK2N1 in prostate cells and clinical specimens was determined by bisulfite sequencing (BS), pyrosequencing, and *in silico* analysis. Results showed that CAMK2N1 was highly methylated in PCa cells and tissues compared to normal prostate epithelial cells and nonmalignant prostate tissues, which was associated with the clinicopathological characteristics in PCa patients. Afterwards, we explored the expression of CAMK2N1 and its DNA methylation level by qRT-PCR, western blot, BS, and methylation-specific PCR in PCa cells after 5-Aza-CdR treatment or DNMT1 genetic modification, which demonstrated that the reduced expression of CAMK2N1 can be restored by 5-Aza-CdR treatment via demethylation. Moreover, DNMT1 formed a positive feedback loop with CAMK2N1 in PCa cells. The expression of CAMK2N1 was downregulated by DNMT1-mediated DNA methylation, which reversely induced DNMT1 expression through activating AKT or ERK signaling pathway. Finally, functional assays including wound healing, invasion, and migration assay, as well as the xenograft model in nude mice indicated that CAMK2N1 inhibited the invasion, migration, and proliferation of PCa cells and these effects were reversed by DNMT1 overexpression. In conclusion, DNMT1-mediated hypermethylation of CAMK2N1 not only downregulates the gene expression but also promotes the progression of PCa.

## 1. Introduction

Prostate cancer (PCa) is the most common malignancy among older males in Western countries, has the second highest mortality rate, and has become a serious global public health problem [1]. The tumorigenesis of PCa is a complex multifactorial and multistep process involving the changes in signaling pathways, oncogenes, and tumor suppressor genes [2, 3]. It is currently believed that the abnormal expression of genes caused by epigenetic modifications, such as DNA methylation, histone acetylation, and noncoding RNAs, plays an important role in the occurrence and development of PCa [4, 5].

Calcium/calmodulin-dependent protein kinase II inhibitor I (CAMK2N1) is an endogenous CaMKII suppressor gene. Recent studies have shown that the Ca^2+^/CaMKII signaling pathway plays an important role in tumorigenesis and that the inhibition of CaMKII blocks tumor cell growth [6, 7]. CAMK2N1 induces apoptotic cell death and inhibits cell proliferation, cell cycle progression, and androgen receptor (AR) expression in PCa [8, 9]. The expression of CAMK2N1 is increased by the activation of PI3K/AKT signaling and decreased in response to androgen signaling [9–11]. Our previous study confirms that CAMK2N1 is significantly downregulated in PCa and negatively correlates with the degree of malignancy [8, 9]. However, the molecular mechanism of CAMK2N1 downregulation in PCa is still unclear.

DNA methylation abnormalities, including hypomethylation or hypermethylation of specific genes, are significant epigenetic changes in PCa [12]. Hypermethylation of CpG islands in promoter region results in downregulated or even inactivated expression of genes such as AR, GSTP1, RASSF1, and APC, which are closely related to the pathological progression of PCa [13–15]. DNA methyltransferase 1 (DNMT1) is one of the important enzymes that catalyzes the process of DNA methylation. Studies have shown that DNMT1 is highly expressed in the nucleus in human PCa specimens and is related to tumor stage, Gleason score, and AR expression [16]. Interference with the expression of DNMT1 restores the expression of certain tumor suppressor genes and suppresses tumor growth [17].

To investigate whether the decreased expression of CAMK2N1 in PCa is associated with DNA methylation and to clarify the potential molecular mechanisms of CAMK2N1 regulation, we conducted a series of experiments. Our findings offer a better understanding of the role of CAMK2N1 DNA methylation in PCa and reveal a promising new approach through which CAMK2N1 and epigenetic pathways could serve as a potential predictive biomarker as well as a molecular determinant in the effective treatment of PCa.

## 2. Materials and Methods

### 2.1. Clinical Samples

A total of 16 benign prostatic hyperplasia (BPH) formalin-fixedparaffin-embedded (FFPE) tissue samples and 52 PCa FFPE tissue samples were randomly collected after surgery at Tongji Hospital, Tongji Medical College, Huazhong University of Science and Technology (HUST). The clinicopathological characteristics of the patients are summarized in Supplementary Table S1. *T* stage shows the size of the tumor, whether it is limited or has spread to the surrounding area. The higher number indicates a larger tumor or more severe effects on the surrounding tissue. *N* stage shows whether tumor has spread to the surrounding lymph nodes. *N*0 indicates no lymph node metastasis and *N*1 indicates that the tumor has metastasized to the lymph nodes. The TNM stage of PCa is a standard way to evaluate how far PCa has spread, which is based on the condition of *T* stage, *N* stage, *M* stage, prostate-specific antigen (PSA) value, and Gleason score according to the information from the American Joint Committee on Cancer (AJCC) [18]. This study was in line with the guidelines of the Declaration of Helsinki, and authorization was obtained from the patients.

### 2.2. Cell Lines and Cell Culture

RWPE-1, LNCaP, DU145, and PC-3 cell lines were purchased from the China Center for Type Culture Collection (CCTCC, China). LNCaP, DU145, and PC-3 cells were maintained in RPMI-1640 medium (HyClone, USA) supplemented with 10% fetal bovine serum (Gibco, USA) and penicillin/streptomycin (Gibco). A defined keratinocyte serum-free medium (Gibco) was prepared for RWPE-1 cell culture. All cell lines were maintained in a humidified incubator at 5% CO_2_ and 37°C.

### 2.3. Antibodies and Chemical Reagents

Antibody details are summarized in Supplementary Table S2. All primary antibodies were diluted according to the supplier's suggestions. 5-Aza-2′-deoxycytidine (5-Aza-CdR), AKTi, and U0126 were purchased from MedChemExpress (USA). PCa cells were exposed to different concentrations of 5-Aza-CdR for 96 h with a daily medium change. All chemical reagents were used according to the manufacturer's instructions.

### 2.4. Plasmid, siRNA, and Cell Transfection

Cloned CAMK2N1 and DNMT1 human cDNA were purchased from Vigene Biosciences (China) and inserted into the pLenti-EF1a-FH-CMV-GFP vector and pAd-EF1a-GFP vector, respectively. Human CAMK2N1 and DNMT1 siRNAs were synthesized as described in Supplementary Table S3. Cells were transfected with plasmids or siRNAs in the presence of Lipofectamine 3000 Reagent (Invitrogen, USA) in Opti-MEM medium (Invitrogen). After 2 or 3 days, cells were harvested for RNA and protein analyses. The lentiviral vectors pGV493-shCAMK2N1 (target seq: 5′- GCAAGCGGGTTGTTATTGA -3′) and pLKO.1-shDNMT1 (target seq: 5′- CGAGTTGCTAGACCGCTTC -3′) were purchased from GeneChem (China) and HedgehogBio Science (China) for plasmid construction, respectively. The constructs were confirmed by sequencing. Then, the plasmids were transfected into HEK293T cells to package the lentivirus used to infect DU145 cells. Cells infected with pGV493-shCAMK2N1 were selected and treated with 2 *μ*g/ml puromycin for 3 days, and cells infected with pLKO.1-shDNMT1 were selected and treated with 6 *μ*g/ml blasticidin for 10 days. Surviving cells were used as stable transfectants.

### 2.5. Publicly Online Database Analysis

An online tool (https://www.ebi.ac.uk/Tools/seqstats/emboss_newcpgreport/) was used to predict the distribution of CpG islands in the promoter region and in the first exon of CAMK2N1 [19]. In this tool, a CpG island is defined as a region of least 200 bp with a proportion of CG dinucleotides exceeding 50% and a ratio of observed/predicted values of CpG higher than 0.6. The promoter is defined as a region starting from 2000 bp (−2000 bp) upstream of the transcription start site (TSS) [20]. TSS refers to the base on the DNA strand that corresponds to the first nucleotide of the mRNA strand, and this position is defined as +1. Transcriptome profiles, methylation profiles, and corresponding clinical information profiles are from the same TCGA_PRAD datasets, which were downloaded from TCGA_Portal (https://portal.gdc.cancer.gov/). CG locus analyzed in the TCGA dataset is the CG dinucleotide that could be methylated, which locates upstream or downstream of CAMK2N1 TSS. We sorted all the transcriptional data, extracted the expression value and corresponding methylation status of CAMK2N1, and merged these with their detailed clinicopathological information.

### 2.6. DNA Isolation, Bisulfite Modification, Methylation-Specific PCR (MSP), Bisulfite Sequencing (BS), Pyrosequencing, and the Genome-Wide DNA Methylation Assay

DNA was extracted using a TIANamp Genomic DNA Kit (Tiangen, China). DNA samples (500 ng) were processed for bisulfite conversion using an EpiTect Fast DNA Bisulfite Kit (Qiagen, Germany) according to the manufacturer's instructions. The bisulfite-modified DNA was used as a template to amplify using an MSP kit (Tiangen). After the reaction, amplification products were examined by agarose gel electrophoresis. As for BS analysis, BS-specific primers were designed according to the CpG islands distribution in the promoter region and the first exon of the CAMK2N1 gene. The bisulfite-modified DNA was amplified by using these four different BS primers. Afterwards, the target fragments in the agarose gel electrophoresis were collected by a DNA Gel Extraction Kit (Generay, China). Subsequently, target fragments were inserted into the pTG19-T vector using a TA cloning kit (Generay). Ten clones were randomly selected and sequenced. There were a total of 22 CG sites in the first BS amplicon of the CAMK2N1 gene. According to it, we designed one pair of PCR primers for pyrosequencing to cover five different CG sites, namely, those at sites 4, 5, 6, 7 and 8. The bisulfite-modified DNA was amplified and PCR was performed according to the instruction of the PyroMark PCR Kit (Qiagen). Single-stranded biotinylated PCR products were purified and subsequently prepared for sequencing with the PyroMark Q24 instrument (Qiagen). The genome-wide DNA methylation percentage was determined with a MethylFlash Methylated DNA Quantification Kit (Colorimetric, Epigentek, USA). The genomic DNA was isolated and bound to strip wells in this assay. The methylated fraction of DNA was detected using antibodies and then quantified colorimetrically by reading the absorbance in a microplate spectrophotometer. The sequences of all primers are shown in Supplementary Table S3.

### 2.7. RNA Isolation and Quantitative Reverse Transcription PCR (qRT-PCR)

The total RNA was isolated from cultured cells using a TRIzol reagent (Invitrogen) according to the established protocol [21]. The total RNA (1 *μ*g) was reversely transcribed to cDNA using PrimeScript RT Master Mix (Takara, China). Then, qRT-PCR was carried out in a Roche Light Cycler 480 system with SYBR Premix Ex Taq TM (Takara). The primer sequences are shown in Supplementary Table S3. Relative gene expression levels were calculated by normalization to GAPDH and quantification via the 2^−△△Ct^ method.

### 2.8. Western Blot

Total protein was isolated from cultured cells with RIPA lysis buffer (Beyotime, China) and 1 mM PMSF buffer (Beyotime). Then, the protein concentration was determined using a BCA protein assay kit (Beyotime). Samples containing equal amounts of protein were loaded into SDS-PAGE gels. After electrophoresis, proteins in the gels were transferred onto PVDF membranes (Millipore, USA). After blocking with 5% bovine serum albumin at room temperature for 2 h, membranes were incubated with the primary antibodies listed above overnight at 4°C. After several TBST washes and incubation with HRP-conjugated secondary antibodies, bound proteins were detected with ECL reagents (Bio-Rad, USA) in a chemiluminescence detection system (Syngene, USA). The bands intensity was quantified with ImageJ software and normalized to that of GAPDH. The relative band intensity in the control group was set to 1.

### 2.9. Immunofluorescence Staining

After washing with phosphate-buffered saline (PBS), cells were fixed with 4% paraformaldehyde for 20 min and permeabilized with 0.1% Triton X-100 for 20 min at room temperature. Then, cells were blocked with goat serum (Beyotime) for 1 h. Afterwards, cells were incubated with primary antibodies at 4°C overnight. After incubation, cells were washed with PBS again and incubated with corresponding secondary antibodies at room temperature for 1 h. Nuclei were stained with DAPI (Beyotime). Slides were mounted and evaluated under an Olympus microscope.

### 2.10. Chromatin Immunoprecipitation (ChIP) Assay

This experiment was performed with a ChIP assay kit (Millipore). Cells were cross-linked with 1% formaldehyde at 37°C for 10 min. Then, cells were lysed for 1 h on ice and sonicated to shear DNA. Anti-DNMT1 ChIP-grade antibody and anti-human IgG antibody were added for incubation at 4°C overnight. Beads were used to precipitate the complexes and enrich the DNA fragments. After multiple washes, cross-linking was reversed by adding 5M NaCl and heating to 65°C for 4 h. Purified DNA was extracted and proceeded to perform PCR analysis. Fold enrichment was calculated by setting the value of the IgG control sample to 1. The sequences of CAMK2N1 are shown in Supplementary Table S3.

### 2.11. Cell Invasion and Migration Assays

For the invasion assay, Matrigel-coated Transwell inserts (Corning, USA) were pretreated with serum-free RPMI-1640 medium at 37°C for 2 h. After removing the medium, we added 750 *μ*l of RPMI-1640 medium supplemented with 10% FBS as a chemoattractant to each lower chamber, added 5 × 10^4^ cells to each upper chamber, and incubated the plates at 37°C for 24 h. Then, the inserts were removed, and noninvaded cells on the upper surface of the membranes were removed with a cotton swab. The invaded cells on the lower surface of the membranes were then fixed with 100% methanol for 15 min and stained with 1% crystal violet. Cells in three microscopic fields were photographed and counted. For the migration assay, a procedure similar to that used in the invasion assay was used, but the Matrigel coating was omitted. Three independent experiments were performed.

### 2.12. Wound Healing Assay

Cells were seeded in a 6-well culture plate and wounds were made with 1-ml pipette tips in the middle of the six-well plates. Then, cells were cultured with serum-free RPMI-1640 medium. After 24 h, cell migration was photographed. The wound coverage area was measured and normalized to that in the 0 h control group to calculate the relative migration rates for comparison. Three independent experiments were performed.

### 2.13. Animal Experiments

BALB/c nude mice (4 weeks old) were purchased from Beijing Vital River Laboratory Animal Technology Co., Ltd (China) and maintained under specific pathogen-free conditions. All animal experiments were approved by the Ethics Committee of Tongji Hospital, HUST. The whole process was carried out in accordance with the “Guide for the Care and Use of Laboratory Animals.” To establish the xenograft tumor model, 1 × 10^6^ DU145 cells were suspended in 100 *μ*l of serum-free RPMI-1640 and implanted subcutaneously into the left axilla of the nude mice. The tumors were measured every week. Five weeks later, the mice were euthanized, and the tumors were weighed. Tumor specimens were fixed and embedded in paraffin for immunohistochemistry analysis.

### 2.14. Immunohistochemistry

This experiment was conducted as described previously [8, 9]. Primary antibodies were used at the appropriate dilutions in the experiments. Sections (4 *μ*m) were prepared from FFPE DU145 tumor tissues harvested from the nude mice.

### 2.15. Statistical Analysis

All results are presented as the mean ± standard deviation (SD) values, and data were analyzed using GraphPad Prism software. At least three repeated experiments were carried out. The statistical significance of differences between the two groups was assessed by using Student's *t*-test. Differences among multiple groups were analyzed using a one-way analysis of variance (ANOVA) followed by Tukey's multiple comparison test. The two-tailed Pearson correlation coefficient was used for correlation analysis. The log-rank test was used to compare survival distributions. Statistical significance was assumed for *P* < 0.05.

## 3. Results

### 3.1. The Promoter of CAMK2N1 Is Hypermethylated in Prostate Cancer Cells Compared to Normal Prostate Epithelial Cells

**Figure 1:**
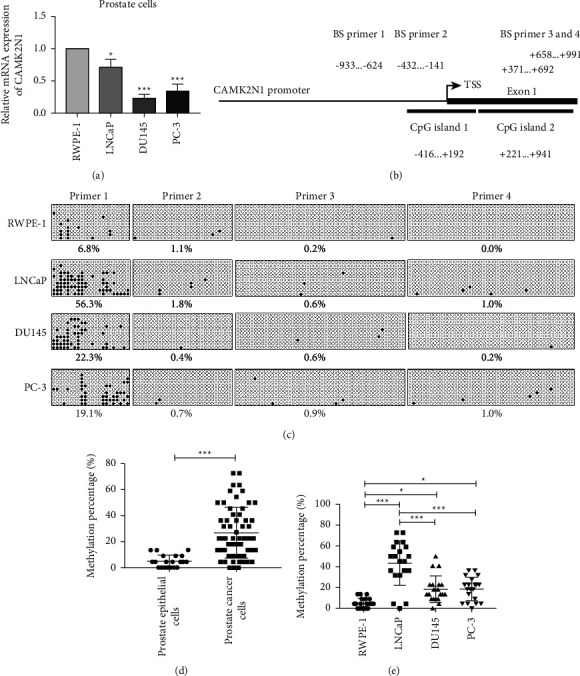
The DNA methylation level of CAMK2N1 in PCa cells and normal prostate epithelial cells. (a) The mRNA expression of CAMK2N1 was determined in RWPE-1, LNCaP, DU145, and PC-3 cells by using qRT-PCR (*n* = 3). (b) The CpG islands distribution of CAMK2N1 in the promoter region and first exon by searching the online tool (https://www.ebi.ac.uk/Tools/seqstats/emboss_newcpgreport/). Four pairs of BS primers were designed to cover these CpG islands of CAMK2N1 in the promoter region and first exon. (c) BS analysis of RWPE-1, LNCaP, DU145, and PC-3 cells. One point represents one CG site, in which black point represents methylated CG site and white point represents unmethylated CG site. One horizontal row represents one clone and one vertical row represents one CG site. Finally, ten clones were selected randomly. (d, e) The quantification of BS analysis at the first amplicon region in normal prostate cells and PCa cells. One point represents the DNA methylation percentage of one clone. There is a total of twenty clones per prostate cell line. Data are presented as mean ± SD, one-way ANOVA test was applied, and ^*∗*^*P* < 0.05 and ^*∗∗∗*^*P* < 0.001 are considered as statistically significant.

We first analyzed the mRNA expression of CAMK2N1 in normal prostate epithelial cells and PCa cells using qRT-PCR. The results showed that CAMK2N1 mRNA expression markedly decreased in LNCaP, DU145, and PC-3 cells compared to RWPE-1 cells, especially in AR-negative DU145 and PC-3 cells (Figure 1(a)).

The enrichment of CG sequences, especially the existence of CpG islands, indicates the possibility of DNA methylation [22]. The publicly available online tool (https://www.ebi.ac.uk/Tools/seqstats/emboss_newcpgreport/) predicted that there were two CpG islands likely to be hypermethylated in the promoter region and in the first exon of CAMK2N1 (Figure 1(b)). We determined the DNA methylation percentage of CAMK2N1 in RWPE-1, LNCaP, DU145, and PC-3 cells through BS. We identified that in the first amplicon, the DNA methylation percentage at these 22 CG sites was significantly higher than that in other amplicons not only in PCa cells but also in normal prostate epithelial cells, indicating that the CG sequences in this region are the key site of methylation for regulating gene expression (Figure 1(c)). The results also showed that the average DNA methylation percentage of CAMK2N1 was 6.8% in RWPE-1 cells but was 56.3% in LNCaP cells, and 22.3% in DU145 cells and 19.1% in PC-3 cells (Figure 1(c)). Based on the abovementioned results, we performed the BS analysis again (Supplementary Figure S1) and quantified the DNA methylation percentage in twenty sequencing clones. We confirmed again that the CG sequences in the CAMK2N1 gene promoter was hypermethylated in prostate cancer cells compared to normal prostate epithelial cells (Figure 1(d)). Interestingly, AR-positive LNCaP cells had a higher DNA methylation percentage than AR-negative DU145 cells and PC-3 cells (Figure 1(e)).

### 3.2. DNA Hypermethylation of CAMK2N1 Is Identified in Prostate Cancer Tissues and Is Associated with Clinicopathological Characteristics

**Figure 2:**
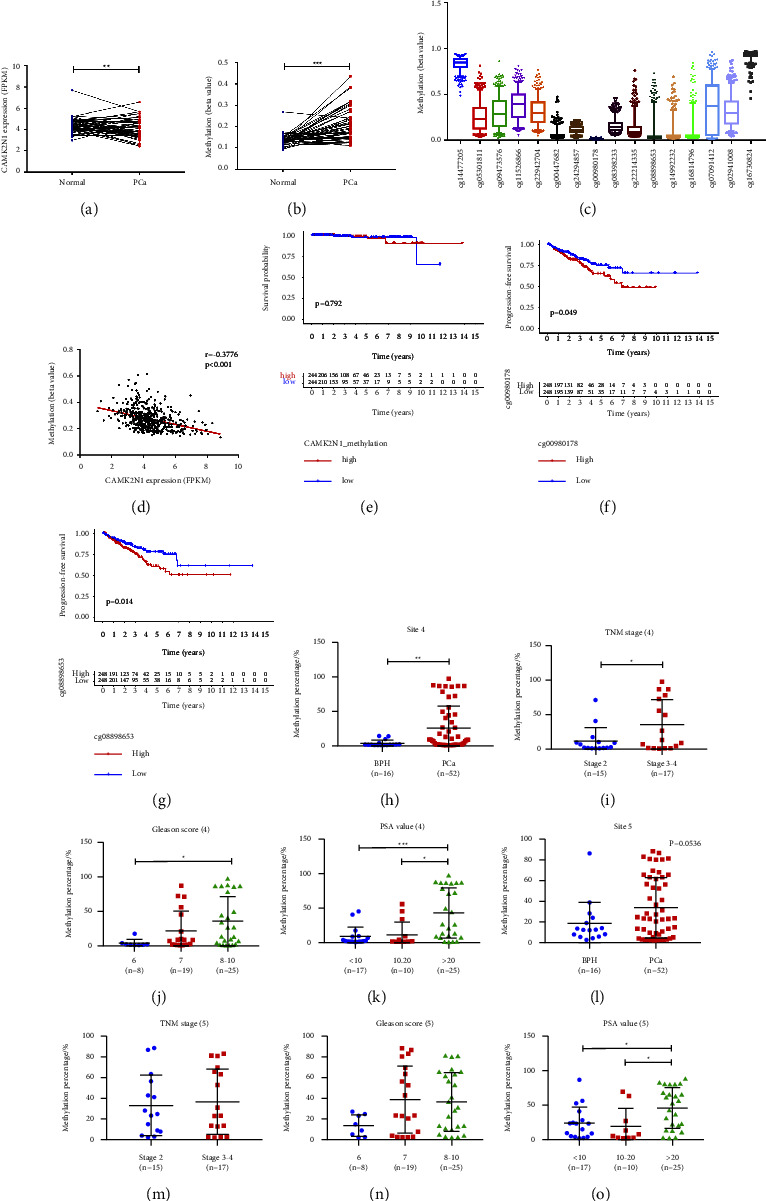
Correlations between DNA methylation, CAMK2N1 expression, and clinical outcomes in PCa tissues. (a) The paired study of CAMK2N1 expression in normal prostate tissues and PCa tissues from TCGA data. (b) The DNA methylation level of CAMK2N1 in normal prostate tissues and PCa tissues from TCGA paired data. (c) The methylation level of CAMK2N1 at all CG loci that were analyzed in TCGA data (plot represents 5–95 percentile). (d) The correlation between the mean value of methylation level of all CG loci in CAMK2N1 gene and gene expression from TCGA data. (e) The overall survival of patients with CAMK2N1 hypermethylation and patients with CAMK2N1 hypomethylation. (f, g) The progression-free survival of PCa patients with different CAMK2N1 methylation levels in CG00980178 locus and CG08898653 locus. (h–k) The quantification of pyrosequencing results at site 4 in BPH and PCa tissues. PCa patients were divided into TNM stage 2 and stage 3–4 groups, Gleason score 6, 7, and 8–10 groups, and PSA value < 10 ng/ml, 10–20 ng/ml, and >20 ng/ml groups (n = 16–52). (l–o) Pyrosequencing results at site 5 (n = 16–52). Data are presented as mean ± SD; unpaired *t* tests, two-tailed Pearson correlation coefficient, one-way ANOVA, and logrank test were used, and ^*∗*^*P* < 0.05, ^*∗∗*^*P* < 0.01, and ^*∗∗∗*^*P* < 0.001 were considered as statistically significant.

To investigate whether there is DNA hypermethylation of CAMK2N1 in PCa tissues, we analyzed data from the TCGA database. The results revealed that CAMK2N1 expression was reduced in PCa tissues compared to normal prostate tissues (Figure 2(a)). Correspondingly, the DNA methylation level of CAMK2N1 in PCa samples was higher than that in normal prostate samples (Figure 2(b)). The CG loci from CG14477205 to CG24294857 were located upstream of TSS, while the other CG loci were located downstream (Figure 2(c)). From the methylation levels of these arrays, compared with the downstream, there was indeed hypermethylation in the upstream of CAMK2N1 TSS, which was consistent with our results in prostate cells shown in Figure 1(c) and Figure 2(c). Moreover, CAMK2N1 gene expression was negatively correlated with the average DNA methylation level for the CG loci (Figure 2(d)). Although CAMK2N1 hypermethylation did not affect the *T* or *N* stages, Gleason scores, PSA values, and the overall survival of PCa patients (Figure 2(e), Supplementary Figure S2A), hypermethylation at certain loci still worsened the progression-free survival of patients (Figures 2(f) and 2(g)).

To further support our hypothesis, we collected FFPE prostate tissues to measure the DNA methylation level of CAMK2N1 between benign samples from 16 BPH patients and tumor samples from 52 PCa patients. According to the location of CAMK2N1 DNA hypermethylation determined in PCa cells, as shown in Figure 1, we elected to use sites 4 to 8 in the first amplicon from BS as the key methylation sites and performed pyrosequencing. The results indicated that at site 4, the DNA methylation percentage of CAMK2N1 in PCa tissues was 7 times higher than that in BPH tissues (Figure 2(h)). At other sites, although there were no significant differences, the DNA methylation percentage in PCa tissues was still higher than that in BPH tissues (Figure 2(l), Supplementary Figures S2B–S2D). Regarding clinical and pathological manifestations, the pyrosequencing results showed that patients with higher DNA methylation percentages of CAMK2N1 at site 4 had higher TNM stages, higher Gleason scores, and higher PSA levels (Figures 2(i)–2(k)). Data for the other sites revealed similar results, although there were no significant differences, patients with hypermethylation of CAMK2N1 showed higher risk, as determined by TNM stages, Gleason scores, and PSA levels (Figures 2(l)–2(o), Supplementary Figures S2B–S2D). Interestingly, we found out that site 4 is the CG22942704 locus that was analyzed in the TCGA dataset. However, TCGA analysis demonstrated that there was no significant difference in methylation level at the CG22942704 locus among PCa patients with different pathological characteristics (Supplementary Figure S2E). Nevertheless, the T4 tumor or Gleason score 10 tumor still has a slightly higher methylation level of CAMK2N1 (Supplementary Figure S2E).

### 3.3. 5-Aza-CdR Restores the Expression of CAMK2N1 through DNA Demethylation

**Figure 3:**
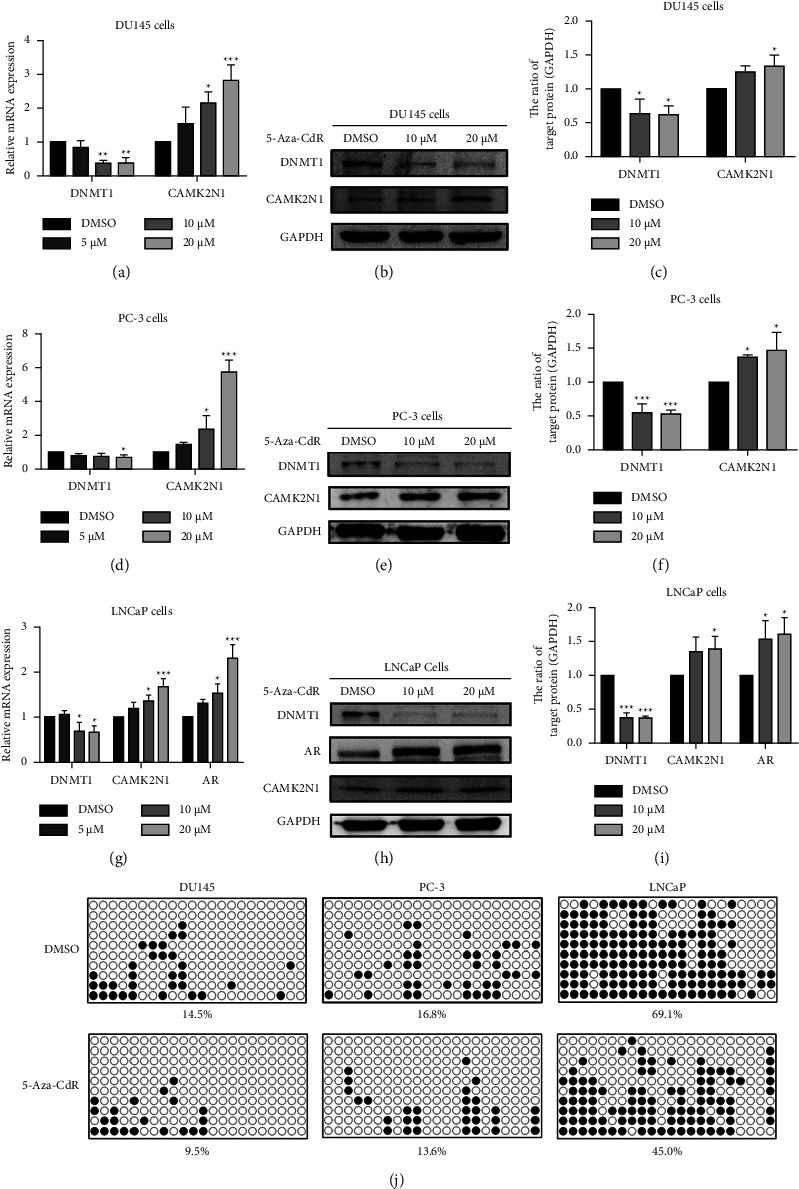
The expression and DNA methylation percentage of CAMK2N1 after 5-Aza-CdR treatment in PCa cells. qRT-PCR analysis was used to determine the effects of 5 *μ*M, 10 *μ*M, and 20 *μ*M 5-Aza-CdR on the mRNA expression of DNMT1 and CAMK2N1 in (a) DU145 and (d) PC-3 cells (*n* = 3). Western blot analysis of CAMK2N1 and DNMT1 expression in (b, c) DU145 and (e, f) PC-3 PCa cells after continuously treating with 10 *μ*M and 20 *μ*M 5-Aza-CdR for 96 h (*n* = 3). (g) The mRNA expression of DNMT1, CAMK2N1, and AR was determined by qRT-PCR in LNCaP cells after 5-Aza-CdR treatment (*n* = 3). (h, i) The protein expression of CAMK2N1, AR, and DNMT1 was analyzed by western blot in LNCaP cells after 5-Aza-CdR treatment (*n* = 3). (j) BS analysis in DU145, PC-3, and LNCaP cells with or without 20 *μ*M 5-Aza-CdR treatment for 96 h. Black and white points represent methylated and unmethylated CG site, respectively. Data are presented as mean ± SD, the one-way ANOVA test was used, and ^*∗*^*P* < 0.05, ^*∗∗*^*P* < 0.01, and ^*∗∗∗*^*P* < 0.001 were considered as statistically significant compared to the DMSO group, and GAPDH was used as a control.

To verify whether DNA methylation is associated with the transcriptional silencing of CAMK2N1 in PCa cells of different subtypes, the DNA methyltransferase inhibitor 5-Aza-CdR was used to induce CAMK2N1 demethylation in PCa cells. qRT-PCR and western blot analyses revealed that 5-Aza-CdR elevated the mRNA and protein expression levels of CAMK2N1 in androgen-independent DU145 and PC-3 cells in a dose-dependent manner (Figures 3(a)–3(f) and Supplementary Figure S3A). DNMT1 expression was inhibited by 5-Aza-CdR (Figures 3(a)–3(f)) and Supplementary Figure S3B). Similarly, in androgen-dependent LNCaP cells, qRT-PCR and western blot analyses showed that 5-Aza-CdR treatment increased the expression of not only CAMK2N1 but also AR, accompanied by the decreased expression of DNMT1 (Figures 3(g)–3(i)). To further clarify the relationships between the expression of CAMK2N1 and its DNA methylation status, the DNA methylation level in PCa cells treated with 5-Aza-CdR were analyzed by BS. The results indicated that the DNA methylation percentage was decreased after 5-Aza-CdR treatment from 14.5% to 9.5% in DU145 cells, from 16.8% to 13.6% in PC-3 cells, and from 69.1% to 45% in LNCaP cells (Figure 3(j)).

### 3.4. DNMT1 Interacts with the Promoter of CAMK2N1 and Inhibits the Expression of CAMK2N1 via DNA Methylation

**Figure 4:**
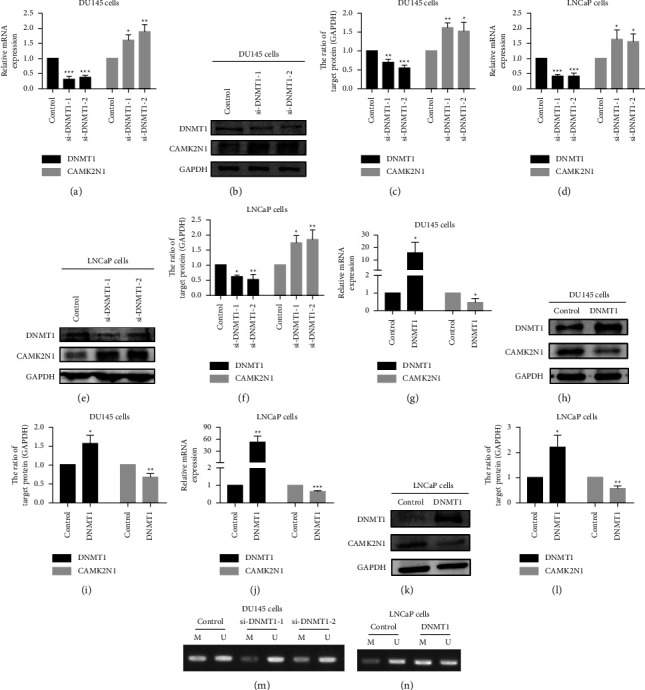
DNMT1 suppresses the expression of CAMK2N1 through DNA hypermethylation in PCa cells. (a–c) DU145 and (d–f) LNCaP cells were transfected with DNMT1 siRNA. After 2 or 3 days, cells were harvested for CAMK2N1 and DNMT1 expression analysis by qRT-PCR and western blot (GAPDH was used as a control; *n* = 3). (g–i) DU145 and (j–l) LNCaP cells were transfected with DNMT1 cDNA clones. After 2 or 3 days, cells were harvested for CAMK2N1 and DNMT1 expression analysis by qRT-PCR and western blot (GAPDH was used as a control; *n* = 3). The representative agarose gel of CAMK2N1 methylated and unmethylated amplicons. After 2 days of treatment of transfection, MSP was performed for DNA methylation analysis in (m) DU145 cells with DNMT1 downregulation and (n) LNCaP cells with DNMT1 upregulation. M means methylated amplicon and *U* means unmethylated amplicon. Data are presented as mean ± SD; the *t*-test and one-way ANOVA test were used, and ^*∗*^*P* < 0.05, ^*∗∗*^*P* < 0.01, and ^*∗∗∗*^*P* < 0.001 were considered as statistically significant compared to the control group.

When we inhibited DNA methyltransferase activity with 5-Aza-CdR, we observed that the expression of DNMT1 was decreased, whereas CAMK2N1 expression was increased. Thus, we hypothesized that DNA methyltransferases, especially DNMT1, may bind to the gene sequence of CAMK2N1 to exert biological effects. To verify this hypothesis, a ChIP assay was performed in DMSO-treated and 5-Aza-CdR-treated DU145 cells. The amplicon of CAMK2N1 that binds to DNMT1 in DMSO-treated DU145 cells can be detected compared to the IgG group (Supplementary Figure S4), indicating that DNMT1 was able to interact with the promoter of CAMK2N1. This conclusion was also supported by the result in another way that the expression of CAMK2N1 combined with DNMT1 was reduced in 5-Aza-CdR-treated DU145 cells compared to DMSO-treated DU145 cells due to the inhibition of DNMT1 caused by 5-Aza-CdR (Supplementary Figure S4). To investigate the specific relationships between DNMT1 and CAMK2N1, we knocked down and overexpressed DNMT1 in DU145 and LNCaP cells. qRT-PCR and western blot analyses showed that suppression of DNMT1 expression increased the mRNA and protein expression levels of CAMK2N1 (Figures 4(a)–4(f)), while overexpression of DNMT1 decreased CAMK2N1 expression (Figures 4(g)–4(l)). The qualitative MSP results showed that decreasing the expression of DNMT1 diminished the methylation status of CAMK2N1, while overexpression of DNMT1 correspondingly enhanced the methylation status of CAMK2N1 in PCa cells (Figures 4(m) and 4(n)).

### 3.5. CAMK2N1 Inhibits the Expression of DNMT1 via the AKT or MER/ERK Signaling Pathway

**Figure 5:**
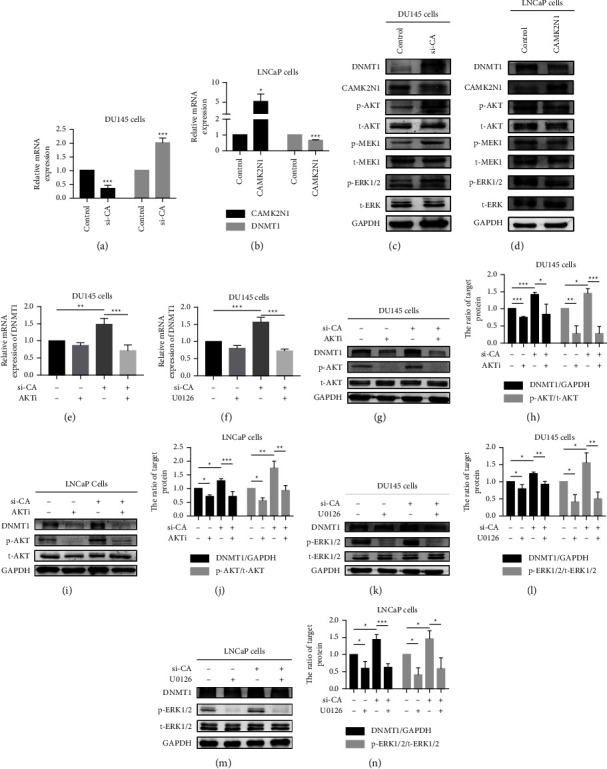
The CAMK2N1-mediated regulation of DNMT1 via the AKT or ERK signaling pathway in PCa cells. (a) DU145 cells and (b) LNCaP cells were transfected with CAMK2N1 siRNA and cDNA clones, respectively. After 2 days, the mRNA expression of DNMT1 and CAMK2N1 was analyzed by qRT-PCR (GAPDH was used as a control; *n* = 3). The expression of CAMK2N1, DNMT1, p-AKT, t-AKT, p-MEK1, t-MEK1, p-ERK1/2, and t-ERK1/2 was determined by western blot in (c) DU145 cells with CAMK2N1 knockdown (si-CA) or (d) LNCaP cells with CAMK2N1 overexpression (GAPDH was used as a control; *n* = 3). CAMK2N1 knockdown DU145 cells were treated with (e) 10 *μ*M AKT signaling pathway inhibitor AKTi or (f) 10 *μ*M ERK signaling pathway inhibitor U0126 for 1 day. The mRNA expression of DNMT1 was analyzed by qRT-PCR (GAPDH was used as a control; *n* = 3). CAMK2N1 knockdown (g, h) DU145 cells and (i, j) LNCaP cells were treated with 10 *μ*M AKTi for 1 day. The protein expression of DNMT1, p-AKT, and t-AKT was analyzed by western blot (GAPDH was used as a control; *n* = 3). CAMK2N1 knockdown (k, l) DU145 cells and (m, n) LNCaP cells were treated with 10 *μ*M·U0126 for 1 day. The protein expression of DNMT1, p-ERK1/2, and t-ERK1/2 was analyzed by western blot (GAPDH was used as a control; *n* = 3). Data are presented as mean ± SD; the *t*-test and one-way ANOVA test were used, and ^*∗*^*P* < 0.05, ^*∗∗*^*P* < 0.01, and ^*∗∗∗*^*P* < 0.001 were considered as statistically significant.

Our group indicated that CAMK2N1 suppresses the PI3K/AKT and MEK/ERK signaling pathways in PCa [8]. Sunahori et al. found that the ERK pathway is an important signaling pathway for DNA methylation regulation and that inhibiting the MEK/ERK signaling pathway reduces the expression of DNMT1 [23]. Therefore, CAMK2N1 may be capable of regulating the expression of DNMT1. Similarly, we downregulated and upregulated CAMK2N1 expression. qRT-PCR and western blot analyses showed that downregulation of CAMK2N1 increased the expression of DNMT1 and induced the phosphorylation-mediated activation of the AKT and MEK/ERK pathways in DU145 cells (Figures 5(a), 5(c)). Conversely, the upregulation of CAMK2N1 showed the opposite effects in LNCaP cells (Figures 5(b), 5(d)). To further prove that CAMK2N1 regulates the expression of DNMT1 through the AKT or ERK pathway, we used the AKT inhibitor AKTi and the ERK inhibitor U0126. The results indicated that in DU145 and LNCaP cells, the knockdown of CAMK2N1 elevated the expression of DNMT1 (Figures 5(e)–5(n)), whereas the addition of the AKT inhibitor (Figures 5(e), 5(g)–5(j)) and ERK inhibitor (Figures 5(f), 5(k)–5(n)) abrogated the effects of CAMK2N1 knockdown on DNMT1 expression not only at the mRNA level but also at the protein level. Although CAMK2N1 can regulate the expression of DNMT1, it cannot affect the genome-wide DNA methylation level by changing DNMT1 expression (Supplementary Figure S5).

### 3.6. Silencing CAMK2N1 Promotes Tumor Progression by Inducing DNMT1 *In Vitro* and *In Vivo*

**Figure 6:**
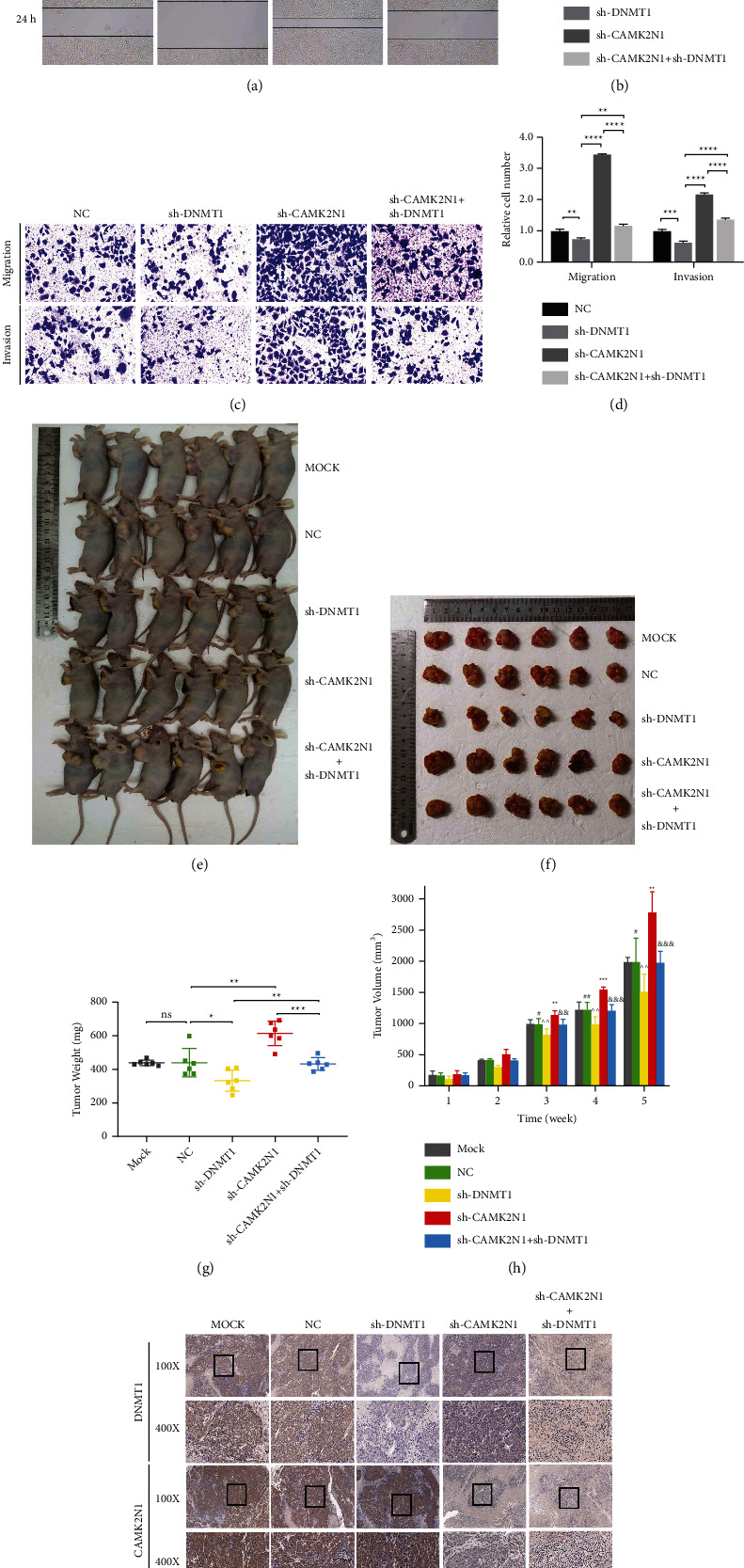
DNMT1 reverses the tumor suppressive effect of CAMK2N1 in PCa cells *in vitro* and *in vivo*. (a, b) The representative figures and the quantification results of wound healing assay. Results were photographed after 24 h incubation. Data were normalized to the 0 h control group (*n* = 3). (c, d) The representative figures and the quantification results of Transwell migration and Matrigel invasion assays. Cells were counted after 24 h incubation. Data were normalized to the negative control group (*n* = 3). (e–h) DU145 cells transfected stably with vector, shDNMT1, shCAMK2N1, or both of shDNMT1 and shCAMK2N1 were injected subcutaneously into the left axilla of BALB/c nude mice. Tumor volume and weight were quantified after five weeks of implantation (*P* value: NC vs. shCAMK2N1: ^*∗∗*^ < 0.01 and ^*∗∗∗*^ < 0.001; NC vs. shDNMT1: ^#^ < 0.05 and ^##^ < 0.01; shCAMK2N1 vs. shCAMK2N1 + shDNMT1: ^&&^ < 0.01 and ^&&&^ < 0.001; shDNMT1 vs. shCAMK2N1 + shDNMT1: ^^^^ < 0.01). (i) Immunohistochemistry analysis of CAMK2N1 and DNMT1 expression in subcutaneous xenograft tumors. 100X and 400X magnification figures were selected. Data are presented as mean ± SD; the *t*-test and one-way ANOVA test were used, and ^*∗*^*P* < 0.05, ^*∗∗*^*P* < 0.01, ^*∗∗∗*^*P* < 0.001, and ^*∗∗∗∗*^*P* < 0.0001 were considered as statistically significant.

To investigate the potential biological effects of DNMT1 and CAMK2N1 on tumor progression, DU145 cell lines with stable knockdown of DNMT1 and CAMK2N1 were established (Supplementary Figure S6). The wound healing assay revealed that the knockdown of CAMK2N1 promoted the migration of DU145 cells, but the knockdown of DNMT1 not only abolished this effect but also inhibited cell migration (Figures 6(a) and 6(b)). Accordingly, the knockdown of CAMK2N1 significantly induced PCa cell migration and invasion in Transwell migration and Matrigel invasion assays, in contrast to the effects of DNMT1 knockdown (Figures 6(c) and 6(d)). These cells were injected subcutaneously into BALB/c nude mice. The tumor volume and weight were significantly increased in the CAMK2N1 knockdown group compared with the control group, while the knockdown of DNMT1 reversed these effects (Figures 6(e)–6(h)). Immunohistochemistry analysis was applied to detect the protein expression of DNMT1 and CAMK2N1 in xenograft tumors of each group. The expression of CAMK2N1 was obviously increased when DNMT1 expression was markedly reduced in tumor tissues (Figure 6(i)). We also found that the knockdown of CAMK2N1 increased the expression of DNMT1, consistent with the results described above.

## 4. Discussion

CAMK2N1 is generally considered as a tumor suppressor gene, which is reduced and associated with poor clinical outcomes in hepatocellular carcinoma, medullary thyroid cancer, cervical cancer, or ovarian cancer [24–27]. In our previous studies, we reported that CAMK2N1 is significantly downregulated in PCa tissues compared to normal and benign prostate tissues, thereby inducing the activation of AR and the activity of PSA [8, 9]. Romaniuk et al. also found that CAMK2N1 expression is significantly reduced in metastatic castration-recurrent PCa tissue compared with androgen-dependent primary tissue [28]. However, little is known about the molecular mechanism of CAMK2N1 downregulation in PCa. Recently, Peng et al. found that DNA hypermethylation contributes to the downregulation of CAMK2N1 in hepatocellular carcinoma, cervical, and ovarian cancer, which serves as a potential prognostic biomarker [24, 27, 29, 30]. Therefore, according to previous valuable literature reports, we hypothesized that DNA methylation may be involved in the abnormal expression of CAMK2N1 in PCa.

BS analysis in PCa cells, *in silico* analysis from the TCGA dataset, and pyrosequencing in PCa tissues confirmed our hypothesis. We found that CAMK2N1 was hypermethylated in PCa cells and PCa tissues compared to nonmalignant prostate cells, normal prostate tissues, and BPH benign tissues. Patients with higher DNA methylation of CAMK2N1 showed higher TNM stages, higher Gleason scores, higher PSA levels, and worst progression-free survival. Notably, our pyrosequencing results validated the TCGA data. The combination of both analyses fully illustrates the presence of hypermethylation of CAMK2N1 in PCa, which could be used as a biomarker. However, due to the limited clinical sample size, we still need to include more patients to confirm it, which is one of the flaws of our study. The degree of DNA methylation was higher in AR-positive LNCaP cells than in AR-negative DU145 and PC-3 cells, which was not consistent with the expression of CAMK2N1 mRNA detected by qRT-PCR. In addition to DNA methylation, there may be other transcriptional modifications or posttranslational modifications that led to the reduced expression of CAMK2N1 in PCa cells, for example, the regulation by transcription factors such as NF-kB, histone acetylation, phosphorylation, or glycosylation. These possibilities require further investigation.

DNMT1 is highly expressed in PCa patients with advanced progression, castration-resistant or metastasis [16]. Loss of DNMT1 is known to be involved in the reactivation of gene expression by demethylation [31]. DNMT1 can repress AR expression in a methylation-dependent or independent manner [32, 33]. In our study, we confirmed by ChIP that DNMT1 binds to the promoter of CAMK2N1. Demethylation drug 5-Aza-CdR as well as DNMT1 knockdown both restored the expression of CAMK2N1 through the DNA demethylation pathway. The expression of AR was also increased in LNCaP cells after 5-Aza-CdR treatment, indicating that there may be methylation modification of the AR gene in PCa. Considering that CAMK2N1 is an androgen-responsive gene [10], there may be some connections among CAMK2N1, androgen/AR pathway, and DNMT1. These possibilities need to be studied further.

Blockade of either AKT signaling or ERK signaling dramatically decreases the expression of DNMT1 in PCa cells [31, 34]. Previous work from our group proved that overexpression of CAMK2N1 inactivates downstream AKT or MEK/ERK signaling pathway [8, 9]. Our work demonstrates that inhibition of CAMK2N1 increased DNMT1 expression and activated the AKT or MEK/ERK signaling pathway. However, when the AKT or ERK signaling pathway was inhibited, the effect of DNMT1 upregulation was abolished. Furthermore, we explored whether the mutual regulation of DNMT1 and CAMK2N1 ultimately affects the progression of PCa *in vitro* and *in vivo*. Inhibition of CAMK2N1 significantly enhanced the migration and invasion of PCa cells and xenograft tumor growth, while the knockdown of DNMT1 reversed these effects. Similarly, the reduction of CAMK2N1 expression also inhibited the tumor suppressive effect of DNMT1 knockdown.

However, growing evidence suggests the opposing role of CAMK2N1 and DNMT1 in PCa. Romanuik et al. showed an elevated level of CAMK2N1 in PCa patients who later had biochemical failure [28]. Furthermore, Carneiro et al. classified CAMK2N1 as an epithelial-mesenchymal transition (EMT)-related gene, which was increased in locally invasive and metastatic PCa [35]. Reduction of DNMT1 promotes PCa metastasis through the induction of EMT, cancer stem cell phenotype, or neuroendocrine differentiation [36, 37]. Considering these contradictory facts, a possible explanation is that the specific expression and function of CAMK2N1 and DNMT1 depend on the stage of PCa. CAMK2N1 and loss of DNMT1 display oncogenic effects via EMT and oncogenes activation caused by genome-wide hypomethylation in the early stage of PCa [35, 38]. In the late stage of PCa, CAMK2N1 expression is decreased and DNMT1 expression is increased, which promotes the further progression of PCa through activation of AR, AKT or ERK signaling pathway, induction of E2F1-mediated proliferation, and inactivation of tumor suppressor genes due to the hypermethylation [8, 9, 24, 28, 38]. Thus, careful evaluation of stages in PCa patients, especially the androgen-dependent situation, is required for fully understanding the effect of CAMK2N1 and DNMT1 on PCa. Moreover, there is little literature to verify that CAMK2N1 does promote the process of EMT in tumors, which could be the focus of our future research.

## 5. Conclusions

**Figure 7:**
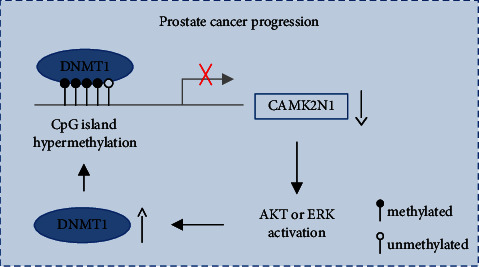
Simplified scheme summarizing the main finding in this study. The levels of CAMK2N1 mRNA expression are downregulated by DNMT1-mediated hypermethylation in the promoter region of CAMK2N1 in PCa. The reduced expression of CAMK2N1 promotes the activation of PI3K/AKT or MEK/ERK signaling pathway, which induces the DNMT1 expression. This positive feedback loop facilitates the progression of PCa *in vitro* and *in vivo*.

Taken together, our results demonstrate that CAMK2N1 is hypermethylated in PCa cells and tissues, resulting in the downregulation of CAMK2N1, which is significantly related to the pathological TNM stages, Gleason scores, and PSA levels in PCa patients. Treatment with 5-Aza-CdR in PCa cells restores CAMK2N1 gene expression by demethylating the promoter of CAMK2N1 in PCa cells. DNMT1 inhibits the expression of CAMK2N1 through DNA methylation, while downregulation of CAMK2N1 increases DNMT1 expression via the AKT or ERK signaling pathway. The positive feedback loop between DNMT1 and CAMK2N1 promotes the progression of prostate cancer *in vivo* and *in vitro* (summarized in Figure 7). In summary, this study highlights that CAMK2N1 and epigenetic pathways could be pathological biomarkers as well as the potential therapeutic targets for the effective treatment of prostate cancer.

## Data Availability

The datasets used and/or analyzed during the current study are available from the corresponding author upon reasonable request.

## Supplementary Materials

Supplementary Materials Table S1: clinicopathological characteristics of the prostate tissue samples. Table S2: list of antibodies used in this study. Table S3: sequences of primers and siRNA (human). Figure S1: BS analysis of RWPE-1, LNCaP, DU145, and PC-3 cells after using BS primer 1. Figure S2: DNA methylation level of CAMK2N1 in the TCGA dataset and PCa tissues. Figure S3: the immunofluorescence staining of CAMK2N1 (Cy3, red) and DNMT1 (FITC, green) in DMSO-treated and 20 *μ*M 5-Aza-CdR-treated DU145 cells. Figure S4: qRT-PCR analysis of CAMK2N1 following ChIP assay was performed to confirm the binding of DNMT1 to the promoter of CAMK2N1 in DMSO-treated and 20 *μ*M 5-Aza-CdR-treated DU145 cells. Figure S5: CAMK2N1 has no effect on the changes of genome-wide DNA methylation level in PCa cells. Figure S6: the validation of shCAMK2N1 and shDNMT1 in DU145 cells.
