# Supplementary material for: Downregulation of CAMK2N1 due to DNA Hypermethylation Mediated by DNMT1 that Promotes the Progression of Prostate Cancer
Source: J Oncol. 2023 Jan 30;2023:4539045. doi: 10.1155/2023/4539045 (PMC9902116; doi:10.1155/2023/4539045)
Supplement: Supplementary Materials — Table S1: clinicopathological characteristics of the prostate tissue samples. Table S2: list of antibodies used in this study. Table S3: sequences of primers and siRNA (human). Figure S1: BS analysis of RWPE-1, LNCaP, DU145, and PC-3 cells after using BS primer 1. Figure S2: DNA methylation level of CAMK2N1 in the TCGA dataset and PCa tissues. Figure S3: the immunofluorescence staining of CAMK2N1 (Cy3, red) and DNMT1 (FITC, green) in DMSO-treated and 20 μM 5-Aza-CdR-treated DU145 cells. Figure S4: qRT-PCR analysis of CAMK2N1 following ChIP assay was performed to confirm the binding of DNMT1 to the promoter of CAMK2N1 in DMSO-treated and 20 μM 5-Aza-CdR-treated DU145 cells. Figure S5: CAMK2N1 has no effect on the changes of genome-wide DNA methylation level in PCa cells. Figure S6: the validation of shCAMK2N1 and shDNMT1 in DU145 cells. [file 4539045.f1.docx]

**Supplementary materials**

**Supplementary Table S1.** Clinicopathological characteristics of the prostate tissue samples.

|  | Prostate cancer | Benign prostatic hyperplasia |
| --- | --- | --- |
| Total number | 52 | 16 |
| Age in years, mean (range) | 67 (53-79) | 69 (60-85) |
| TNM stage* |  |  |
| T2, n | 15 |  |
| T3, n | 8 |  |
| T4, n | 9 |  |
| Gleason scores |  |  |
| 6, n | 8 |  |
| 7, n | 19 |  |
| 8-10, n | 25 |  |
| PSA values ng/ml, mean (range) | 53.13 (0.008-647) |  |
| <10, n | 17 |  |
| 10-20, n | 10 |  |
| >20, n | 25 |  |

***: TNM stages were not available for all patients.**

**Supplementary Table S2.** List of antibodies used in this study.

| **Antibodies** | **Companies** | **Catalog No.** | **Dilutions** |
| --- | --- | --- | --- |
| Rabbit polyclonal anti-CAMK2N1 | Sigma-Aldrich, USA | SAB1302411 | 1:500 (WB), 1: 100 (IF, IHC) |
| Rabbit monoclonal anti-DNMT1 | Abcam, UK | Ab188453 | 1:1000 (WB), 1:500 (IF), 1:100 (IHC) |
| Mouse monoclonal anti-DNMT1 | Novus Biologicals, USA | NB100-56519 | 1:100 (ChIP) |
| Rabbit polyclonal anti-AR | Affinity Biosciences, USA | AF6137 | 1:500 (WB) |
| Mouse monoclonal anti-Akt | Cell Signaling Technology (CST), USA | 2920 | 1:1000 (WB) |
| Rabbit monoclonal anti-Phospho-Akt (Ser473) | CST | 4060 | 1:1000 (WB) |
| Rabbit monoclonal anti-MEK1 | CST | 12671 | 1:1000 (WB) |
| Rabbit monoclonal anti-Phospho-MEK1 (Thr292) | CST | 26975 | 1:1000 (WB) |
| Rabbit monoclonal anti-ERK1/2 | CST | 4695 | 1:1000 (WB) |
| Rabbit monoclonal anti-Phospho-ERK1/2 (Thr202/Tyr204) | CST | 4370 | 1:1000 (WB) |
| Rabbit polyclonal anti-GAPDH | Affinity Biosciences | AF7021 | 1:5000 (WB) |
| Goat anti-rabbit IgG (H+L) HRP | Affinity Biosciences | S0001 | 1:5000 (WB) |
| Goat anti-mouse IgG (H+L) HRP | Affinity Biosciences | S0002 | 1:5000 (WB) |

**Supplementary Table S3.** Sequences of primers and siRNA (human).

| **Gene** | **Forward Sequence** | **Reverse Sequence** |
| --- | --- | --- |
| **qRT-PCR** |  |  |
| GAPDH | GCACCGTCAAGGCTGAGAAC | TGGTGAAGACGCCAGTGGA |
| CAMK2N1 | TGCAGGACACCAACAACTTC | GCACGTCATCAATCCTATCATC |
| AR | GGCAAGAGCACTGAAGATACT | TGTAGAGAGACAGGGTAGACG |
| DNMT1 | GCAAGATTGTGGTGGAGTTCC | ATGGGCTGCTCATCACTGTC |
| **BSP** |  |  |
| Primer 1 | GAGGGTAGGTGTTGTTGGTT | AAAAAAAAAAACRCCTCATTAC |
| Primer 2 | TGGGGAGGAAGGGTTTTT | ACTACCRCTAACTTCCTACCCC |
| Primer 3 | TYGTAGTTTTTAGTTTGTTTTTTYGAAT | ACCRAAAAACRCCRCTAAAA |
| Primer 4 | GTGTYGYGTTTTTGTTTT | TACTCCRACCRATCTAACC |
| **Pyrosequencing** |  |  |
| Primer | TTGTGGTTAGGTAGGTTTTATTAAGTAAAG | ATCTCCCTTCCAACAACAAC |
| **MSP** |  |  |
| CAMK2N1 (M) | AATTAGGAGGGGACGTTAAAATC | ACATATATCCCTAACAAACAACGAA |
| CAMK2N1 (U) | GAATTAGGAGGGGATGTTAAAATT | ACATATATCCCTAACAAACAACAAA |
| **ChIP** |  |  |
| CAMK2N1 | CGCGTGTGCGTCGTCTGTC | GCTCACGGGTGTTTACGGG |
| **siRNA** |  |  |
| DNMT1-1 | CGAGUUGCUAGACCGCUUC TT | GAAGCGGUCUAGCAACUCG TT |
| DNMT1-2 | CGGUGCUCAUGCUUACAAC TT | GUUGUAAGCAUGAGCACCG TT |
| CAMK2N1 | GCAAGCGGGUUGUUAUUGA TT | UCAAUAACAACCCGCUUGC TT |

**R=A or G; Y=C or T**

**Supplementary Fig. S1**


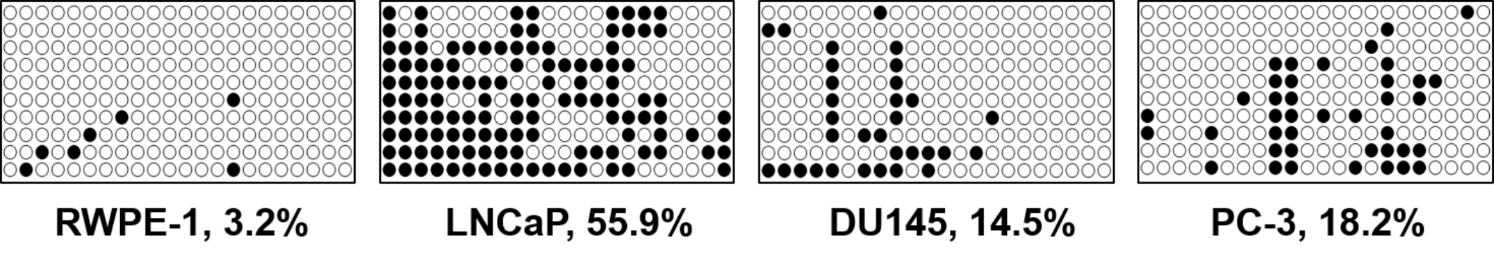


**Supplementary Fig. S1** BS analysis of RWPE-1, LNCaP, DU145 and PC-3 cells after using BS primer 1. One point represents one CG locus, in which black point represents methylated CG site and white point represents unmethylated CG site. One horizontal row represents one clone and one vertical row represents one CG site. Finally, ten clones were selected randomly.

**Supplementary Fig. S2**


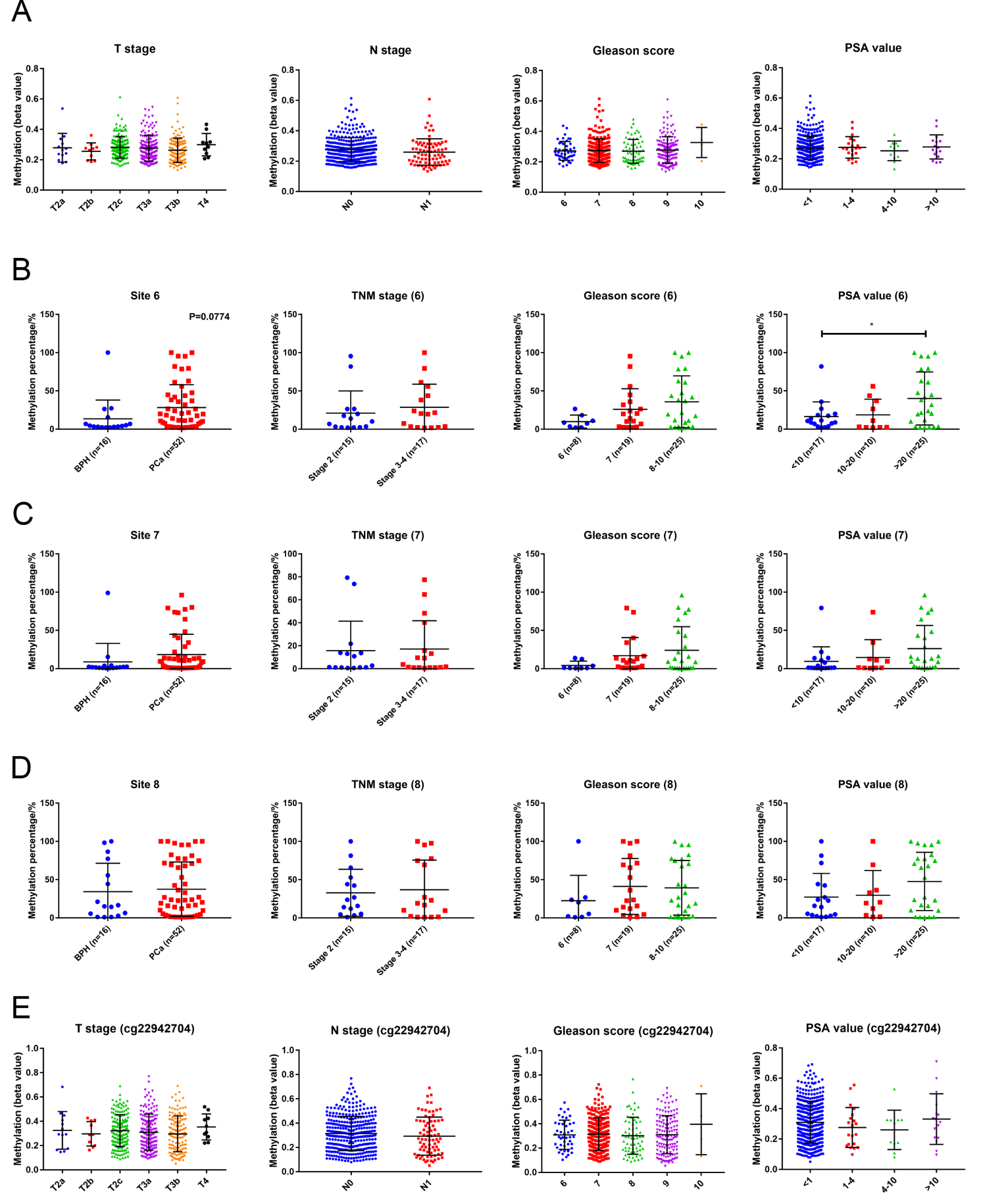


**Supplementary Fig. S2** DNA methylation level of CAMK2N1 in TCGA dataset and PCa tissues. **A** DNA methylation level of CAMK2N1 in PCa patients with different T or N stages, Gleason scores and PSA values from TCGA database. **B** The quantification of pyrosequencing results at site 6 in BPH and PCa tissues. PCa patients were divided into TNM stage 2 and stage 3-4 groups, Gleason score 6, 7 and 8-10 groups, PSA value < 10 ng/ml, 10-20 ng/ml and > 20 ng/ml groups (n=16-52). **C** Pyrosequencing results at site 7. **D** Pyrosequencing results at site 8. **E** DNA methylation level of CAMK2N1 at cg22942704 locus in PCa patients with different T or N stages, Gleason scores and PSA values from TCGA database. Data are presented as mean ± SD, t test, one-way ANOVA test and log rank test were used, *P < 0.05.

**Supplementary Fig. S3**


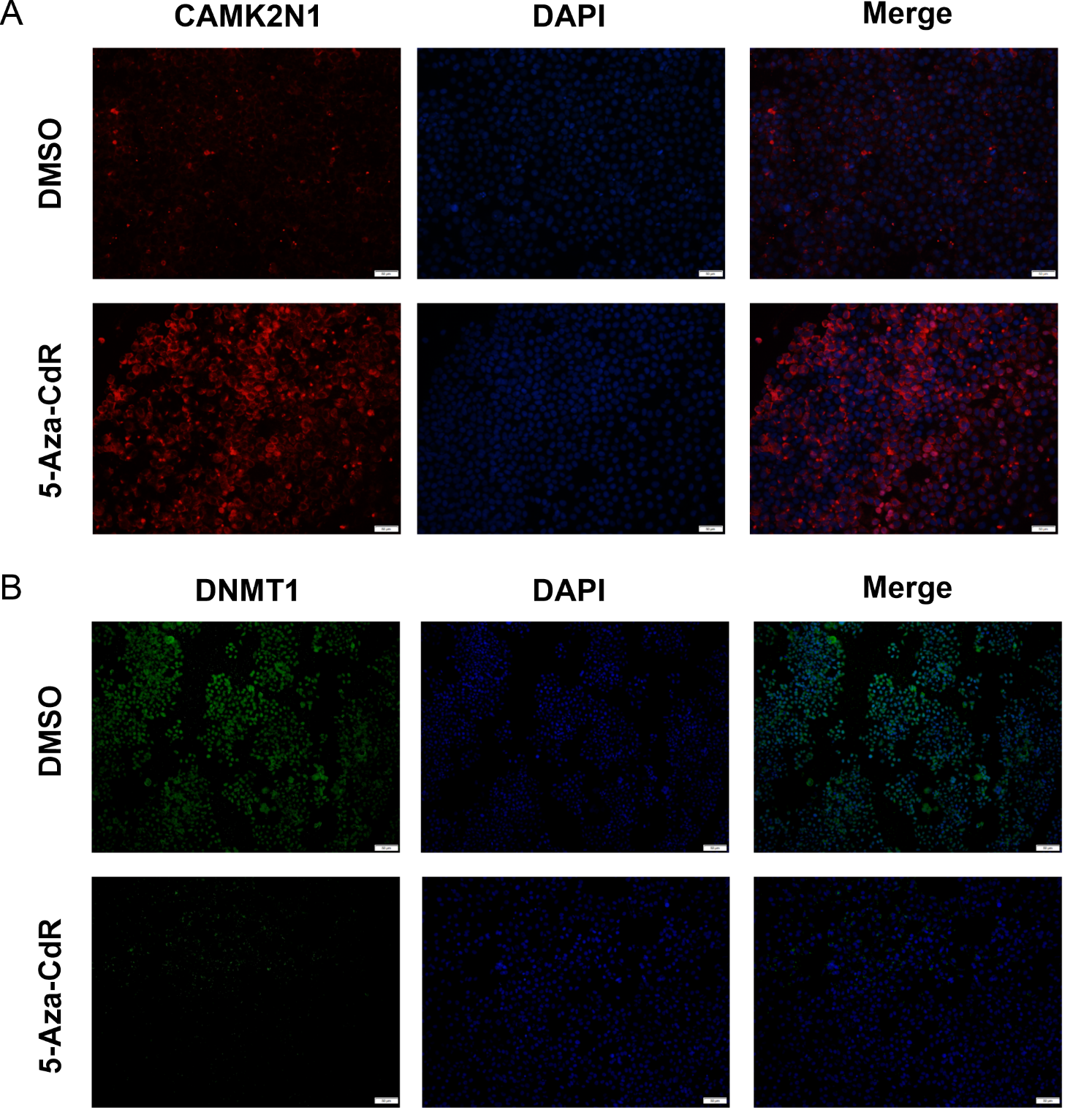


**Supplementary Fig. S3** The immunofluorescence staining of **A** CAMK2N1 (Cy3, red) and **B** DNMT1 (FITC, green) in DMSO-treated and 20 μM 5-Aza-CdR-treated DU145 cells. The nuclei were stained with DAPI. Scale bars represent 50 μm.

**Supplementary Fig. S4**


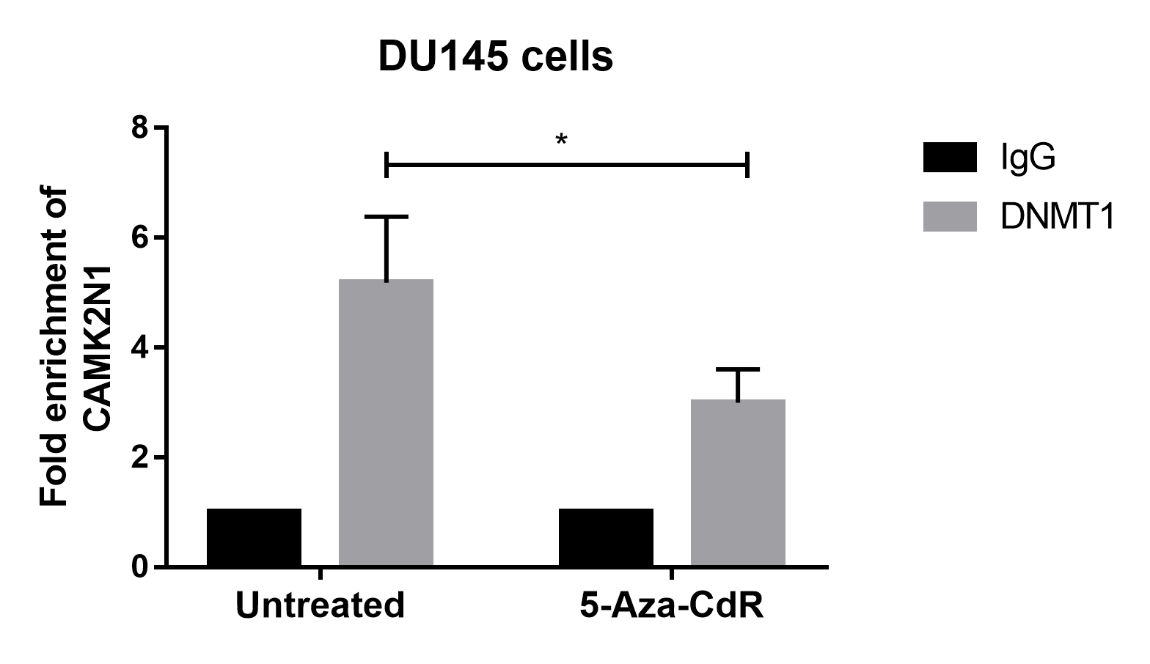


**Supplementary Fig. S4** qRT-PCR analysis of CAMK2N1 following ChIP assay was performed to confirm the binding of DNMT1 to the promoter of CAMK2N1 in DMSO-treated and 20μM 5-Aza-CdR-treated DU145 cells. Data were normalized to input DNA and displayed as fold enrichment relative to IgG group. IgG antibody, input DNA and blank control were used as controls (n=3). Data are presented as mean ± SD, t test was used, *P < 0.05.

**Supplementary Fig. S5**


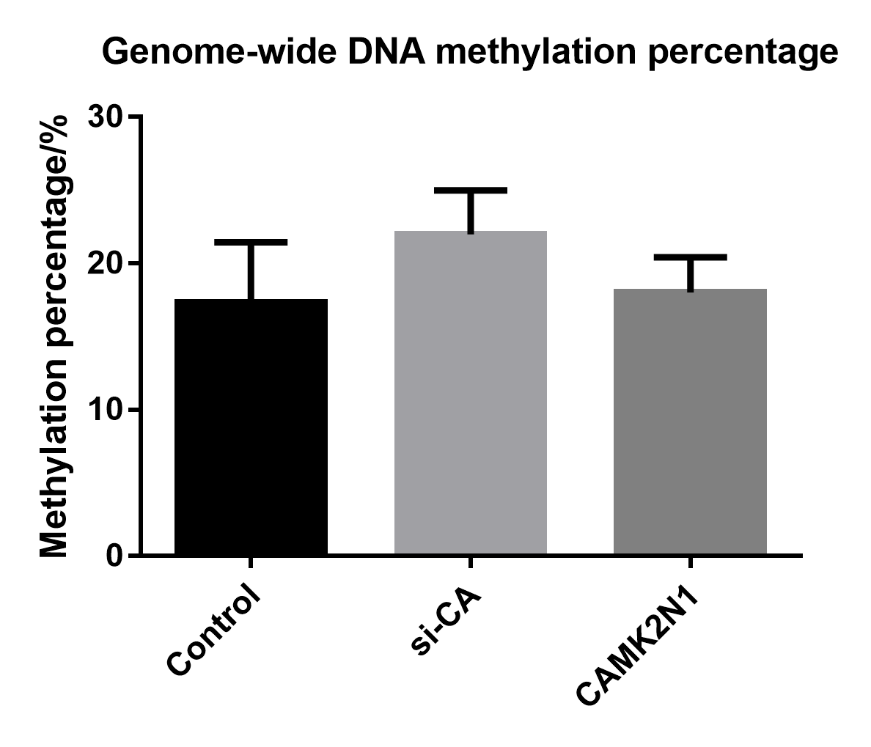


**Supplementary Fig. S5.** CAMK2N1 has no effect on the changes of genome-wide DNA methylation level in PCa cells. DU145 cells were transfected with CAMK2N1 siRNA or cDNA clones. After 2 days, DNA was extracted and the genome-wide DNA methylation level was assessed by a Methylated DNA Quantification Kit (n=6-7). Data are presented as mean ± SD, one-way ANOVA test were used.

**Supplementary Fig. S6**

**
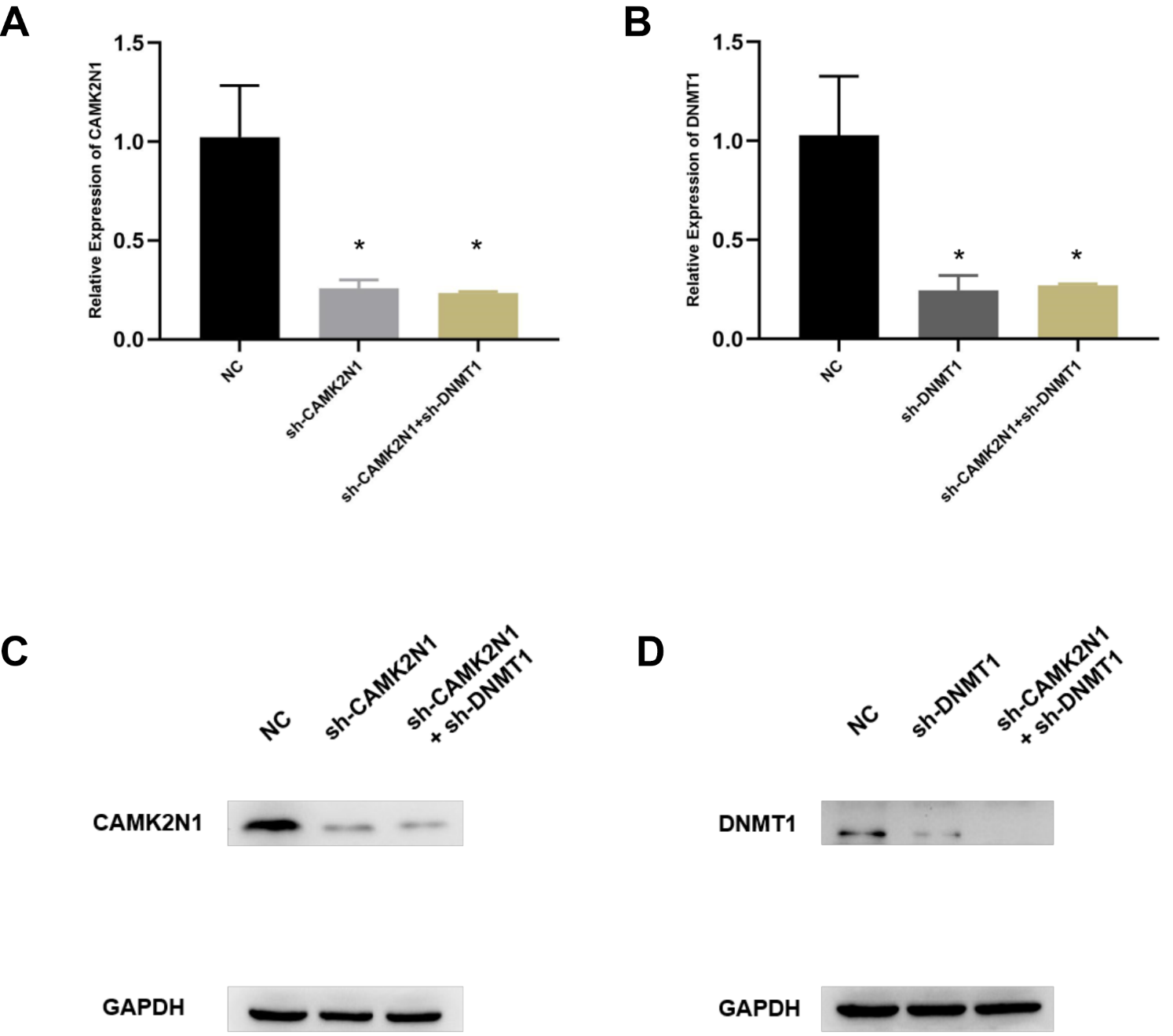
**

**Supplementary Fig. S6.** The validation of sh-CAMK2N1 and sh-DNMT1 in DU145 cells. The mRNA expression of **A** CAMK2N1 and **B** DNMT1 was analyzed by qRT-PCR in sh-CAMK2N1 and sh-DNMT1 DU145 cells respectively, as well as in NC and double knockdown DU145 cells (n=3). The protein expression of **C** CAMK2N1 and **D** DNMT1 was analyzed by western blot in NC, sh-CAMK2N1, sh-DNMT1 or double knockdown DU145 cells. Data are presented as mean ± SD, one-way ANOVA test was used, *P < 0.05, compared to the NC group.
